# Premature Pubarche: Time to Revise the Diagnostic Approach?

**DOI:** 10.3390/jcm12062187

**Published:** 2023-03-11

**Authors:** Federico Baronio, Alice Marzatico, Rosaria De Iasio, Rita Ortolano, Antonio Fanolla, Giorgio Radetti, Antonio Balsamo, Andrea Pession, Alessandra Cassio

**Affiliations:** 1 Department Hospital of Woman and Child, Pediatric Unit, IRCCS AOU di Bologna Policlinico di S.Orsola, 40138 Bologna, Italy; 2 Metropolitan Laboratory, AUSL Bologna, 40124 Bologna, Italy; 3 Observatory for Health Provincial Government, 39100 Bolzano, Italy; 4 Marienklinik, 39100 Bolzano, Italy; 5 Department of Medical and Surgical Sciences, University of Bologna, 40126 Bologna, Italy

**Keywords:** non-classic congenital adrenal hyperplasia, early pubarche, 17OH Progesterone, bone age, ACTH test

## Abstract

Premature pubarche (PP) could represent the first manifestation of non-classic congenital adrenal hyperplasia caused by 21 hydroxylase deficiency (NC21OHD) (10–30% of cases). In the last 20 years, the necessity of performing an ACTH test to diagnose NC21OHD in all cases with PP has been questioned, with conflicting results. This study aims to retrospectively evaluate the predictive value of the basal androgens, 17-OHP levels, and auxological features in suggesting the presence of NC21OHD and, thus, the need for a standard ACTH test to confirm the diagnosis. In all, 111 consecutive patients (87 females) with PP and advanced bone age underwent an ACTH test. Of these, 6/111 cases (1 male) were diagnosed with NC21OHD. The mean baseline 17 hydroxyprogesterone (17-OHP), dehydroepiandrosterone (DHEA), dehydroepiandrosterone sulfate (DHEA-S), delta 4 androstenedione (Δ4A), and testosterone serum levels were higher in NC21OHD patients than in the others (*p* < 0.05). We found three predictive features for NC21OHD: basal 17 OHP of >200 ng/mL, bone age advance of >2 years, and DHEA-S levels of >228 ng/mL with sensitivity and specificity of 83.3% and 97.1%, 83.3% and 65.7%, and 83.3% and 96.2%, respectively. Our data confirm that the prevalence of NC21OHD is low among patients with PP. Serum 17-OHP of >200 ng/mL could be helpful to decide, in most cases, which patients should undergo the ACTH test. Bone age advance represented an inadequately specific predictive marker of NC21OHD.

## 1. Introduction

Adrenarche is a physiological condition that usually occurs after five years of age due to the maturation of the adrenal cortex (zona reticularis): it is characterized by the rising of adrenal androgens dehydroepiandrosterone (DHEA) and DHEA sulfate (DHEA-S) [1]. The clinical manifestation of adrenarche is called pubarche, which consists of the appearance of pubic hair, axillary hair, adult body odor, and acne. Pubarche is considered premature if it appears before the age of eight years in girls and nine years in boys. In 70–90% of cases, premature pubarche (PP) simply represents an anticipation of the prepubertal physiologic adrenarche; in this case, it is called idiopathic premature pubarche or premature adrenarche (PA), and it is characterized by the elevation of serum DHEA-S from 1.08 μMol/L (40 μg/dL) to 3.5 μMol/l (130 μg/dL) [1]. Premature pubarche could also be a manifestation of an increased sensitivity of the hair follicle to normal androgen levels, and in this case, it is generally called “isolated premature pubarche” (IPP) [2]. In patients with PA or IPP, stature, bone age, and growth velocity are not affected; however, some cases show significant bone age advancement and growth acceleration [3,4,5,6]. In the other 10–30% of cases, PP is the first manifestation of non-classic congenital adrenal hyperplasia due to mild 21 hydroxylase deficiency (NC21OHD), an autosomal recessive genetic condition due to homozygous or compound heterozygous variants on *CYP21A2*. The biochemical marker of the disease is represented by adrenal hyperandrogenism with increased levels of 17 hydroxyprogesterone (17-OHP) and delta 4 androstenedione (Δ4A) [7]. NC21OHD, if left untreated, leads to accelerated growth velocity, early puberty, reduced adult height, irregular menses, hirsutism, and acne later in adolescence and adulthood [8,9].

The measurement of serum androgens and 17-OHP by the 250 μg Synachten test (standard ACTH test) has been classically utilized to discriminate children with NC21OHD among cases with premature pubarche. The diagnosis of NC21OHD is made by plotting the stimulated 17-OHP levels against the basal values in the nomogram created by New et al. [10]; stimulated 17-OHP above 30 nmol/l (1000 ng/dL) has 100% diagnostic sensitivity and specificity [8,10,11,12,13,14]. Final diagnostic confirmation of NC21OHD should be made by molecular analysis of *CYP21A2*.

Although in clinical practice, in patients with PP, the standard ACTH test is still often performed, it is also well known that in a large proportion of cases, its use is “unnecessary”, as up to 80% of patients with PP are not affected by NC21OHD [15]. The test’s continued use mainly depends on the difficulty of finding predictive clinical markers of NC21OHD, which many authors have researched in the last decades, with conflicting results.

Due to these uncertainties, even now, in our Centre, the clinical and laboratory management of children with PP is cautious and well-consolidated. Once a rapid and severe progression of hyperandrogenism has been excluded by clinical and anamnestic evaluation, for which specific investigation is promptly started, the patient undergoes a hand X-ray: if their bone age is at least one year ahead of their chronological age, the patient undergoes a standard ACTH test to exclude NC21OHD.

This study aims to retrospectively evaluate the predictive value of the basal androgens, 17-OHP levels, and auxological features in suggesting the presence of NC21OHD and, thus, the need for a standard ACTH test to confirm the diagnosis.

## 2. Materials and Methods

We evaluated clinical, radiological, and laboratory data of all children who were referred to our Centre between January 2017 and July 2020 who, according to the diagnostic protocol of the Centre, underwent standard ACTH testing for premature pubarche associated with advanced bone age of at least one year with respect to chronological age.

We collected information about perinatal history (gestational age, neonatal weight) and auxological parameters at the first clinical evaluation. Height was measured by using a Harpenden stadiometer (Holtain Ltd., Crymych, UK; accuracy of 0.1 cm), weight was measured by using a steelyard scale (with an accuracy of 0.1 kg), and BMI was calculated by the formula (weight (kg)/height^2^ (m)); anthropometric parameters (height, weight, BMI) were normalized by age and sex according to the Italian standards of Cacciari et al. [16] and expressed as the standard deviation score (SDS); and clinical data about hyperandrogenism (age at pubarche onset as reported by the patient or her/his parents) and pubertal development (Tanner stage of pubic and axillary hair, testicular volume in males by Prader orchidometer, and breast button in females) were recorded.

The patients born with body weight or length below −2 SDS were defined as small for gestational age (SGA).

The standard ACTH test (250 mg of Synachten i.v.) with a measurement of baseline and 60 min serum 17-OHP was performed in all cases to exclude NC21OHD due to 21 hydroxylase deficiency [17,18]. During the standard ACTH test, the baseline and after-stimulus levels of the following androgens other than cortisol and 17-OHP are usually measured at our Center: DHEA, DHEA-S, and Δ4A. The level of Δ4A was evaluated using an immunochemiluminescence (CLIA) commercial kit Immulite 2000-XPi, while DHEA-S was assessed using the immunochemiluminescence (CLIA) method via Access DXI 800 (Beckman Coulter^®^ Brea, California, USA). DHEA and 17-OHP were assessed using radioimmunological assay kits DSL-9000 and kit DSL-5000, respectively. Basal testosterone levels were also evaluated using the immunochemiluminescence (CLIA) method via Access DXI 800 (Beckman Coulter^®^ Brea, California, USA). Bone age (BA) was estimated by hand X-ray utilizing the method of Greulich and Pyle by experienced pediatric radiologists at our Centre [19].

ACTH-stimulated 17-OHP of >30 nmol/l (1000 ng/dL) was considered to be diagnostic for NC21OHD [11]; otherwise, those with basal serum DHEA-S levels of >40 mcg/dL were considered affected by premature adrenarche (PA), and others without PA or NC21OHD were labeled as idiopathic premature pubarche (IPP) [10,20,21].

All patients with stimulated 17-OHP of >30 nmol/l (1000 ng/dL) underwent diagnostic confirmation by mutational analysis of the CYP21A2 gene.

### Statistical Analysis

To explore the data, preliminary analyses were performed. Continuous data are presented as the mean (SD) or with 95% CIs. Mean values were tested for statistical significance using 2-tailed t-tests. Pearson correlation coefficients were calculated to assess the relationship between test indexes. ANOVA analysis was performed to compare group means for each test index, and the Bonferroni test was used for multiple comparisons. Receiver operating characteristic (ROC) curves were then generated to obtain the values of the area under the curve (AUC) with 95% CIs, sensitivity, and specificity. In addition, the likelihood ratio (LR+ and LR-) and positive and negative predictive values (+PV and -PV, respectively) were also examined. Adjusted ROC analysis using clinical cut-points was performed to identify the best predictor for each index. To determine the optimal cut-off, the Youden index was calculated. The significance threshold was set at *p* < 0.05. The data were analyzed using SAS Enterprise Guide 4.3 (SAS Institute Inc., Cary, NC, USA).

## 3. Results

During the study period, 111 children underwent standard ACTH tests for premature adrenarche associated with advanced bone age: 87 (78.4%) were female, and 24 (21.6%) were male. Five out of the eighty-seven females were already on therapy with GnRH analogue for early central puberty at the time of the ACTH test. The clinical data of the patients are reported in Table 1.

At the time of the ACTH test, the most frequently encountered symptom was pubic hair (84 patients, 75.6%), followed by axillarche (45 patients, 40.5%) and adult body odor (37 patients, 33.3%), variably associated.

### 3.1. Adrenal Steroid Evaluation

Patients were subdivided into three groups: 15/111 cases (2 males) were defined as affected by IPP (Group 1); 90/111 cases (21 males) were diagnosed with PA (Group 2); and 6/111 cases (1 male) were diagnosed with NC21OHD (Group 3) (Table 2).

The mean baseline 17-OHP, Δ4A, testosterone, DHEA, and DHEA-S serum levels were significantly higher in Group 3 than in Groups 1 and 2 (*p* < 0.05), and baseline testosterone was also significantly higher in Group 2 than in Group 1 (*p* < 0.05) (Table 2).

### 3.2. Anthropometric Parameters and Bone Age

The anthropometric features evaluated (height SDS, weight SDS, BMI SDS, age at pubarche onset) and mean delta BA-CA did not significantly differ among Groups 1–3. Patients with IPP underwent standard ACTH tests significantly earlier than the others (Table 2).

To evaluate whether the severity of bone age advancement correlates with basal androgen and 17-OHP levels, the patients were subdivided into three groups: group A, 59/111 patients (53%) with BA-CA of 1 to 2 years; group B, 41/111 patients (37%) with BA-CA of 2 to 3 years; and group C, 11/111 patients (10%) with BA-CA of ≥3 years. The mean baseline 17-OHP, Δ4A, and DHEA-S were significantly higher in patients of group C than in those of groups A and B, whereas mean baseline testosterone was higher in patients of group C than in those of group A (*p* < 0.05) (Table 3).

Five out of six patients (83%) with NC21OHD at the time of the ACTH test showed bone age advancement of more than two years.

### 3.3. Puberty

None of the subjects showed clinical signs of pubertal development (enlarged testicular volume of >4 mL or Tanner stage B2), except for five patients who were on GnRH analogue at the time of ACTH test. Three out of these five patients turned out to also be affected by NC21OHD. One showed basal 17 OHP of 112 ng/mL with a bone age advance of 1.4 years.

### 3.4. NC21OHD Patients

The clinical features of the NC21OHD patients are reported in Table 4. The diagnosis of NC21OHD was confirmed in these patients by mutational analysis of the CYP21A2 gene.

## 4. Statistical Correlations

Basal 17-OHP, Δ4A, testosterone, and DHEA-S strongly correlated with each other and with stimulated 17-OHP levels. Bone age advance (BA-CA) positively correlated with basal 17-OHP, Δ4A, testosterone, and stimulated 17-OHP.

When patients with basal 17-OHP serum levels of >1000 ng/mL were excluded from the analysis (Pearson’s analysis), basal Δ4A positively correlated with other basal hormone levels (17-OHP, DHEA, DHEA-S, testosterone), testosterone positively correlated significantly with basal Δ4A and DHEA-S, and only basal 17 OHP and Δ4A positively correlated with stimulated 17-OHP levels. Bone advance (BA-CA) did not correlate with basal or stimulated 17-OHP and androgens.

**Figure 1:**
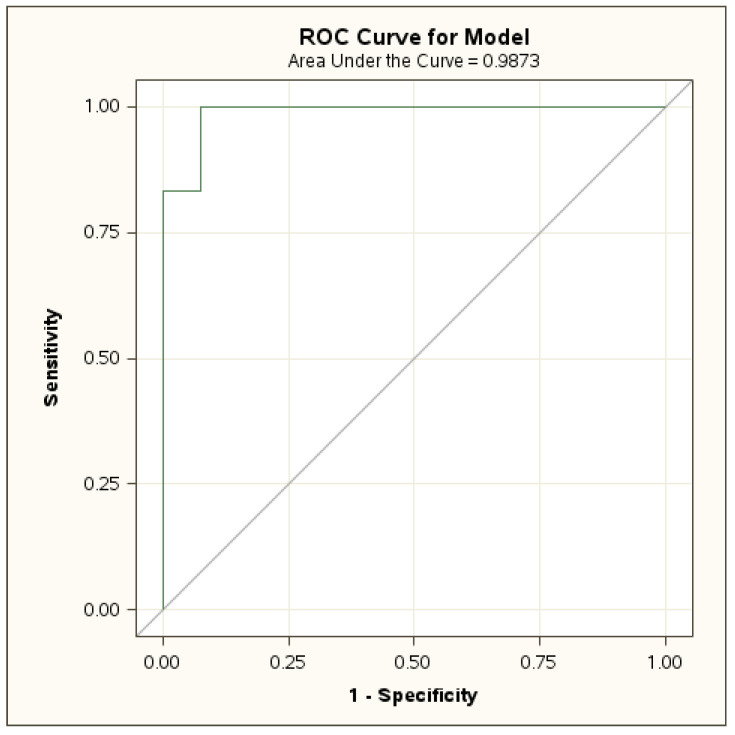
ROC curve and area under the curve (AUC) for basal 17-OHP.

In Table 5, the sensitivity, specificity, negative likelihood ratio (NLR), positive likelihood ratio (PLR), negative predictive value (NPV), and positive predictive value (PPV) of basal and stimulated 17-OHP, basal Δ4A, DHEA-S, testosterone, and bone age advance (BA-CA) are reported. Figure 1 reports the ROC curve and AUC for basal 17-OHP.

In our analysis, we also found a cut-off for DHEA-S of 228 ng/dL with 83% sensitivity and 96% specificity (AUC 0.9) and another for bone advance of ≥2 years with 83% sensitivity and 66% specificity (AUC 0.7) in predicting NC21OHD. The positive and negative predictive values of the DHEA-S basal threshold were 55.6 and 99%, respectively, while for bone age advance, they were 12.2 and 98.6%, respectively. The AUC did not show any statistical differences.

**Figure 2:**
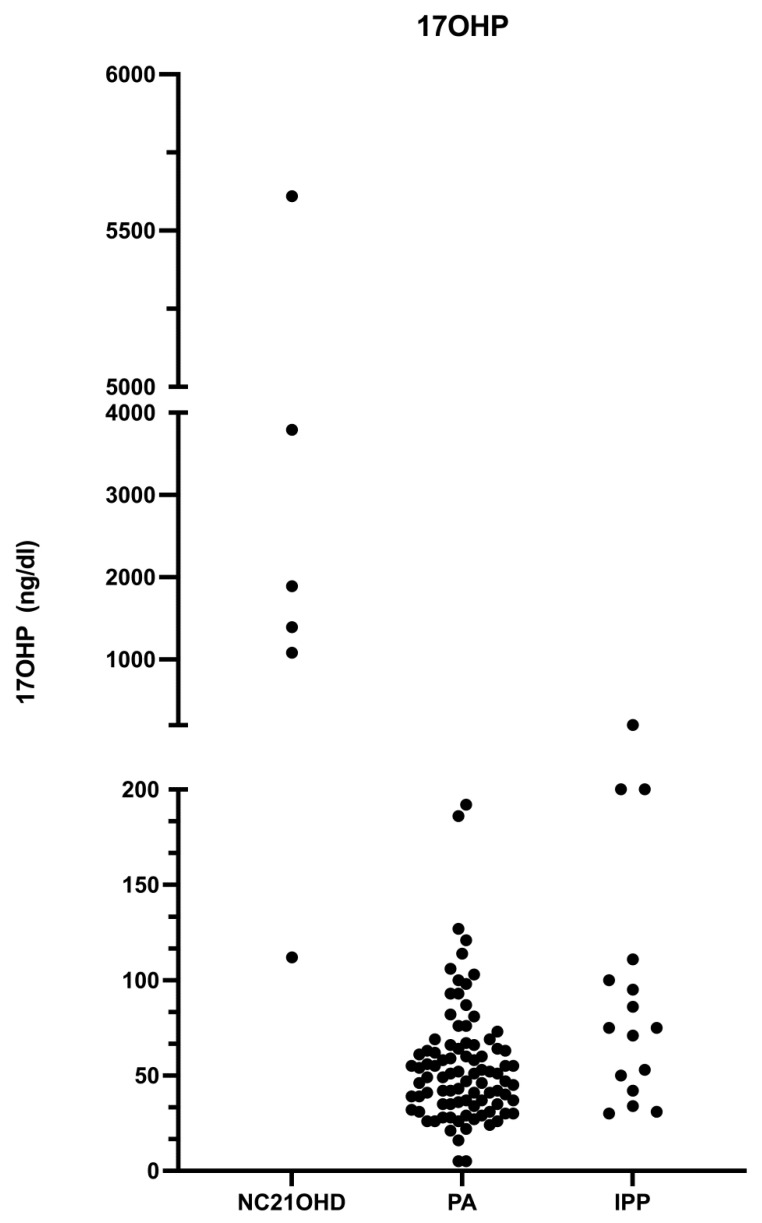
Basal 17-OHP levels in patients subdivided by diagnosis.

**Figure 3:**
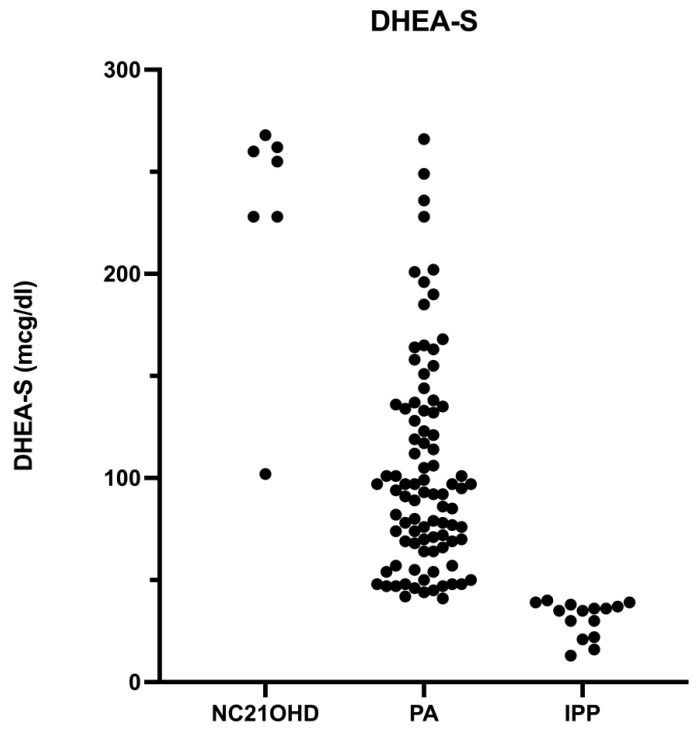
Basal DHEA-S levels in patients subdivided by diagnosis.

For basal 17-OHP, DHEA-S (Figure 2 and Figure 3), and testosterone, it was not possible to find a threshold with 100% specificity and sensitivity due to the presence of hormonal overlap between NC21OHD and PA patients.

## 5. Discussion

In our cohort, the prevalence of NC21OHD was low (5.4% of cases), with a large proportion of cases showing PA (81%) or IPP (13.6%). Our results confirm that, a posteriori, the ACTH test was unnecessary in more than 90% of cases. These results are in line with several research papers published in the last 20 years [7,15,20,21]. To reduce the use of this time-consuming, stressful, and expensive procedure, it seems reasonable to follow the more recent CAH guidelines [17], which indicate that it is possible to exclude or diagnose NC21OHD without performing an ACTH test for 17-OHP levels of <200 ng/mL and >1000 ng/mL, respectively. The same guidelines suggest performing ACTH testing in cases with intermediate basal 17-OHP levels (200–1000 ng/dL) [17]. We agree that in a large proportion of our patients with premature pubarche, basal 17-OHP of <200 ng/mL could have been sufficiently accurate to exclude, per se, the diagnosis of NC21OHD [15,21]. In our series, a basal 17-OHP level of <200 ng/dL represented a threshold with 99% negative predictive value. However, we must underline that in one NC21OHD case, baseline 17-OHP serum levels were 112 ng/dL; therefore, without the ACTH test, we would have missed the diagnosis. In this case, the early pubertal onset associated with elevated DHEA-S serum levels represented two significant clinical and biochemical findings useful to support the decision to perform the standard ACTH test. In agreement with other authors, we did not find any other secure biochemical predictors of NC21OHD [22]. A DHEA-S threshold of 228 ng/dL showed good sensitivity and specificity but low predictive positive value and evident overlap between NC21OHD and other patients. Moreover, we could not find a significant difference between NC21OHD patients and the others with PP with regard to height SDS, BMI SDS, or the onset of symptoms. This is in accordance with other authors, such as Armengaud et al., who found no clinical feature predictive of NC21OHD among cases with PP apart from slight overweight [15]. This is partially in contrast with Bizzarri et al., who found that patients with NC21OHD were thinner, with an earlier onset of symptoms compared to the other cases with precocious pubarche [23]. These findings are probably due to differences in cohort size or local cultural and environmental factors. NC21OHD should be investigated in case of early puberty associated with PP due to the high frequency of diagnoses in this group (three cases out of five). We are not surprised by these results because sustained hyperandrogenism can lead to maturation of the hypothalamus–pituitary–gonadal axis in prepubertal children, as reported by Neeman et al., who showed a significant prevalence of central precocious puberty (4.8%) in a ten-year cohort of 147 girls with NC21OHD [24]. In our Centre, bone age evaluation is often performed as the first examination test in patients with PP as the first step in the diagnostic workup. Nearly all our cases with NC21OHD showed significantly advanced bone maturation (mean BA-CA, 2.7 years; range, 1.4–3.4). Bone age advance of >2 years is a sensitive feature (83%) but with relatively low specificity (67%). As reported by other authors, bone advance in PP could be considered a common and benign occurrence in most cases and should not prompt, per se, an invasive diagnostic workup [4].

## 6. Conclusions

Our data confirm the low prevalence of NC21OHD in patients with PP and the difficulty of finding effective clinical predictive markers of the disease. A 17-OHP basal threshold of 200 ng/mL could be considered a safe and helpful cut-off for clinicians to decide which patients should undergo the ACTH test. Bone age advance represented an inadequately specific predictive marker of NC21OHD in our PP patients. Early central puberty associated with PP should be considered a robust clinical suggestion to exclude NC21OHD carefully. The strengths of this study consist in the absence of selection bias in our cases due to the uniformity of criteria in the decision to perform the ACTH test. The main weakness of our report mainly consists of its retrospective nature.

## Figures and Tables

**Table 1:** Clinical and anthropometric features at first evaluation [mean (SD)]. NS: Non-Significant.

	Males (n.24)	Females (n.87)	
Gestational age (wks)	38 (3.5)	39 (2.5)	NS
Neonatal weight (gr)	2829 (375)	3140 (643)	NS
Chronological age (yrs)	8.9 (1.5)	7.9 (1.0)	*p* < 0.01
Bone age (yrs)	10.8 (1.7)	9.7 (1.2)	*p* < 0.01
Delta BA-CA (yrs)	2.1 (0.6)	2.0 (0.6)	NS
Height SDS	0.9 (0.9)	0.9 (1.0)	NS
BMI SDS	0.4 (1.0)	0.7 (0.8)	NS
Pubarche onset (yrs)	7.6 (1.6)	6.7 (1.0)	*p* < 0.05

**Table 2:** Mean (SD) androgen levels, clinical features, and bone age advancement of patients subdivided by diagnosis. NS: Not Significant.

	Group 1 IPP (n = 15)	Group 2 PA (n = 90)	Group 3 NC21OHD (n = 6)	PA vs. IPP	NC21OHD vs. IPP	NC21OHD vs. PA
Baseline 17-OHP (ng/dL)	89.6 (61.6)	55.3 (31.9)	2312 (2022)	NS	*p* < 0.05	*p* < 0.05
Stimulated 17-OHP (ng/dL)	331.2 (239.6)	236.1 (104.3)	4923 (1604)	NS	*p* < 0.05	*p* < 0.05
Baseline Δ4A (ng/dL)	33.4 (19.6)	56.9 (42.0)	374 (145)	NS	*p* < 0.05	*p* < 0.05
DHEA-S (mcg/dL)	31.1 (8.9)	103.3 (51.3)	229 (64)	NS	*p* < 0.05	*p* < 0.05
Testosterone (ng/mL)	0.06 (0.04)	0.2 (0.2)	0.7 (0.3)	*p* < 0.05	*p* < 0.05	*p* < 0.05
Height SDS	0.7 (0.7)	1 (0.9)	0.6 (1.4)	NS	NS	NS
Weight SDS	0.7 (0.6)	0.8 (0.8)	0.5 (1)	NS	NS	NS
BMI SDS	0.3 (0.6)	0.7 (09)	0.3 (1.4)	NS	NS	NS
BA-CA (yrs)	2.3 (0.9)	1.9 (0.6)	2.6 (0.8)	NS	NS	NS
Age at symptoms	6.3 (1.3)	7.0 (1.2)	6.9 (0.7)	NS	NS	NS
Age at test	7.2 (1.1)	8.3 (1.2)	8.9 (0.9)	*p* < 0.05	*p* < 0.05	NS

**Table 3:** Basal and stimulated mean (SD) 17-OHP, Δ4A, and DHEA-S levels among patients grouped by BA advance. NS: Not Significant.

Groups (BA-CA)	A	B	C	B vs. A	C vs. A	C vs. B
	(1 < BA-CA < 2)	(2 ≤ BA-CA < 3)	(3 ≤ BA-CA)			
	n = 64	n = 37	n = 10			
Baseline 17-OHP (ng/dL)	58.2 (42.9)	172 (398)	1008 (1993)	NS	*p* < 0.05	*p* < 0.05
Baseline Δ4A(ng/dL)	53.3 (47.8)	77.8 (77)	157 (212)	NS	*p* < 0.05	*p* < 0.05
DHEA-S (mcg/dL)	93.4 (51.3)	108.4 (72.6)	115.3 (84.4)	NS	*p* < 0.05	*p* < 0.05
Testosterone (ng/mL)	0.16 (0.2)	0.20 (0.2)	0.33 (0.4)	NS	*p* < 0.05	NS
No. of pts with NC21OHD	1	3	2	-	-	-

**Table 4:** Clinical features and androgen levels in patients with NC21OHD.

Pt	Sex	CA yrs at ACTH Test	Height SDS	BMI SDS	BA-CA (yrs)	Early Puberty	Baseline Testosterone (ng/mL)	Baseline 17-OHP (ng/dL)	Stimulated 17-OHP (ng/dL)	Baseline Δ4A (ng/dL)	Baseline DHEA (ng/mL)	Baseline DHEA-S (mcg/dL)
1	F	9.3	−1.2	1.3	1.4	Yes	0.5	112.0	3270	291.0	10.8	262.0
2	F	9.4	−0.4	1.5	2.9	Yes	0.7	1080.0	3380	319.0	15.3	255.0
3	M	10.3	0.3	−1.3	2.1	Yes	0.6	1390.0	5340	214.0	6.0	268.0
4	F	7.7	0.6	−1.0	2.5	No	0.5	1890.0	7410	323.0	4.9	102.0
5	F	8.7	1.6	−0.6	3.4	No	1.2	3790.0	4250	605.0	17.2	260.0
6	F	8.4	2.7	1.7	3.2	Yes	0.9	5610.0	5890	494.0	18.0	228.0

**Table 5:** Sensitivity, specificity, positive likelihood ratio (PLR), negative likelihood ratio (NLR), positive predictive value (PPV), and negative predictive value (NPV).

	Baseline 17-OHP (ng/dL)		Stimulated 17 OHP (ng/dL)	Δ4A (ng/dL)	Testosterone (ng/mL)	DHEA-S (ng/dL)	BA-CA (Years)
Cut-off	111	200	3271	214	0.4	228	2
Sensitivity (%)	100	83.3	100	100	100	83.3	83.3
Specificity (%)	92.4	97.1	100	100	94.3	96.2	65.7
PPV (%)	42.9	62.5	100	100	50	55.6	12.2
NPV (%)	100	99	100	100	100	99	98.6
PLR	13.1	29.2	-	-	17.5	21.9	2.4
NLR	0	0.17	-	-	0	0.2	0.3
AUC	98.7	-	100	100	97.8	93.7	74.3

## Data Availability

Data are available on request due to restrictions on privacy.
